# Surgical Optimization in Preoperatively Low-risk cN1a PTC: A Predictive Model for High-Volume Central Lymph Node Metastasis

**DOI:** 10.1245/s10434-025-18569-y

**Published:** 2025-10-22

**Authors:** Yi Zhou, Zhixin Guo, Jianyan Long, Heyang Xu, Mingwei Liang, Yuan Hu, Ruixia Li, Zhenbang Ke, Wanna Chen, Xiangdong Xu

**Affiliations:** 1 Department of Thyroid and Breast Surgery, Sun Yat-sen University First Affiliated Hospital, Guangzhou, China; 2 National Clinical Research Center for Cancer, Cancer Hospital Chinese Academy of Medical Sciences Shenzhen Center, Shenzhen, China

**Keywords:** Papillary thyroid carcinoma, Central lymph node, Thyroid surgery, Nomogram

## Abstract

**Background:**

Accurate preoperative identification of high-volume central lymph node metastasis (hv-CLNM; defined as more than 5 central lymph node metastases) is critical for guiding surgical decisions—lobectomy or total thyroidectomy—in patients with papillary thyroid carcinoma (PTC) clinically diagnosed with central neck lymph node metastasis (cN1a). Total thyroidectomy is generally preferred for patients with hv-CLNM. In contrast, lobectomy may be sufficient for patients with low-volume metastasis (5 or fewer lymph node metastases). This study aimed to identify predictors of hv-CLNM in preoperatively low-risk cN1a and to develop a predictive model to estimate the risk of hv-CLNM, thereby optimizing surgical decision-making.

**Methods:**

A total of 707 patients with pathologically confirmed PTC and classified as preoperatively low-risk cN1a were retrospectively enrolled. Clinical and ultrasound features were collected. Variables were selected using least absolute shrinkage and selection operator regression, followed by multivariate logistic regression to construct a predictive model. Internal validation was performed. Recurrence-free survival was compared between lobectomy and total thyroidectomy groups using propensity score matching.

**Results:**

Hv-CLNM occurred in 13.4% (96/707) of patients. Independent predictors of hv-CLNM included age, sex, tumor size, tumor location, and lymph node calcification. The nomogram demonstrated good discrimination (area under the plasma concentration-time curve = 0.75) and calibration. After adjustment, recurrence-free survival did not significantly differ between surgical groups.

**Conclusions:**

This nomogram, based on readily available clinical and ultrasound features, effectively predicts the risk of hv-CLNM in preoperatively low-risk cN1a PTC. This tool may facilitate individualized surgical planning. Lobectomy appears to be a safe and appropriate option for most patients in this subgroup.

**Supplementary Information:**

The online version contains supplementary material available at 10.1245/s10434-025-18569-y.

Papillary thyroid carcinoma (PTC) is the most common form of thyroid malignancy, with a 10-year survival rate exceeding 90%.^1,2^ Despite its generally indolent nature, PTC frequently metastasizes to cervical lymph nodes, particularly within the central compartment, significantly influencing therapeutic decision-making.^3^

Total thyroidectomy removes all thyroid tissue, enabling effective postoperative radioactive iodine (RAI) ablation and more accurate monitoring through serum thyroglobulin (Tg) levels. However, it necessitates lifelong thyroid hormone-replacement therapy. In contrast, lobectomy preserves residual thyroid function in approximately 30–50% of patients and significantly lowers the risk of surgical complications, such as permanent hypoparathyroidism and recurrent laryngeal nerve injury. As a result, lobectomy is increasingly preferred for patients with low-risk PTC.^4,5^

Recent guidelines advocate more conservative, individualized approaches to avoid overtreatment in low-risk cases.^6^ National Comprehensive Cancer Network Clinical Practice Guidelines in Oncology panel members recommend lobectomy for patients with PTC who have incidental low-volume pathologic N1a metastases (fewer than 5 involved nodes with no metastasis >2 mm, in largest dimension).^7^ These recommendations reflect a broader shift toward risk-adapted, lower-intensity treatment strategies.

Among the key factors in risk stratification, lymph node metastasis plays a critical role.^8,9^ A high metastatic lymph node burden (more than 5 metastatic lymph nodes) is strongly associated with higher recurrence rates and poorer prognosis.^2^ Consequently, in the 2015 American Thyroid Association Management Guidelines for Adult Patients with Thyroid Nodules and Differentiated Thyroid Cancer, lymph node metastasis was formally incorporated as a key prognostic factor. Specifically, the presence of more than 5 metastatic lymph nodes is classified as an intermediate-risk feature, with an estimated structural disease recurrence rate of approximately 20%. In contrast, patients with 5 or fewer metastatic lymph nodes are considered low-risk, with a recurrence rate of around 5%.^10^ Radioactive iodine ablation is not routinely recommended for patients with low-risk features but should be considered for intermediate-risk cases based on individual risk stratification and clinical judgment.

Ultrasound is the most widely used imaging modality for preoperative thyroid evaluation. In low-risk patients, suspicious central lymph node metastasis often leads to total thyroidectomy. This is because total thyroidectomy allows for postoperative RAI therapy if high-volume central lymph node metastasis (hv-CLNM) is confirmed by postoperative pathology. However, ultrasound has limited sensitivity for detecting central lymph node metastases (0.284; 95% confidence interval [CI] 0.270–0.298).^11^ It also cannot accurately assess the number of metastatic nodes. In patients with 5 or fewer lymph node metastases, RAI is generally not recommended. Therefore, the need for contralateral thyroid lobe removal in these patients has become increasingly controversial. It is clinically important to assess the true proportion of patients who actually harbor hv-CLNM, and whether such cases can be accurately predicted preoperatively based on clinical and ultrasound features.

This study addresses the critical clinical need for preoperative prediction of central lymph node metastatic load in patients with PTC who are clinically diagnosed with central neck lymph node metastasis (cN1a). By developing a precision assessment model to discriminate between ‘5 or fewer’ and ‘more than 5’ metastatic central lymph nodes, we aim to optimize surgical strategy selection, preserving thyroid function and reducing the risk of procedure-related complications through lobectomy in low-risk cohorts while ensuring adequate intervention for those at intermediate to high risk.

## Materials and Methods

This research employed a single-center retrospective design, and all participating patients were anonymized. Hence, this research was permitted by the research ethics committee and the informed consent requirement was waived. We retrospectively analyzed the medical records of 14,435 patients with PTC who underwent thyroidectomy at The First Affiliated Hospital of Sun Yat-sen University from January 1, 2014, to September 30, 2024.

The inclusion criteria were: (1) adults aged ≥18 years, (2) patients newly diagnosed with PTC without prior treatment, (3) single malignant nodule confined to one thyroid lobe on preoperative ultrasound, (4) preoperative ultrasound indicates lymph node metastasis limited to the central compartment, and (5) underwent at least unilateral or bilateral central compartment lymph node dissection.

The exclusion criteria were: (1) presence of distant metastasis; (2) tumor size >4 cm; (3) evidence of extracapsular extension on preoperative imaging; (4) history of neck surgery, radiation exposure, or thermal ablation; and (5) family history of thyroid cancer.

**Fig. 1:**
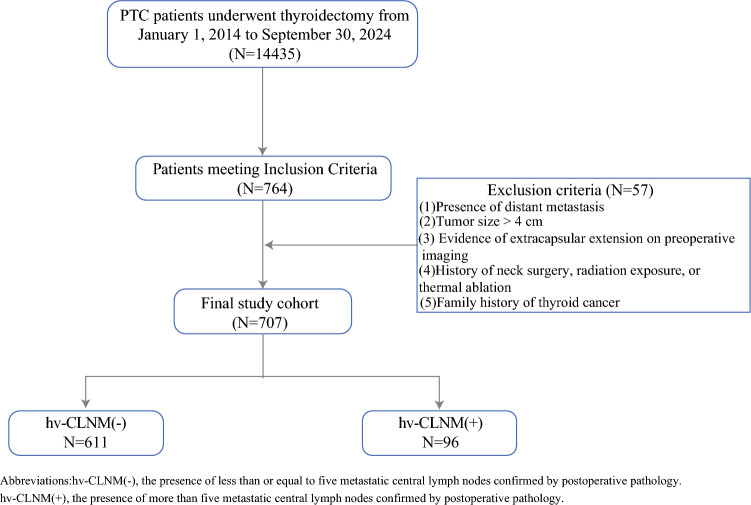
Flowchart of enrolled patients with cN1a papillary thyroid carcinoma (PTC). hv-CLNM(-), the presence of five or fewer metastatic central lymph nodes confirmed by postoperative pathology; hv-CLNM(+), the presence of more than five central lymph nodes confirmed by postoperative pathology

A total of 707 patients were ultimately included in the analysis (Fig. 1). Among them, 96 patients were classified as having hv-CLNM, defined as the presence of more than 5 metastatic central lymph nodes confirmed by postoperative pathology.

### Patient Clinical Characteristics and Preoperative Ultrasonographic Assessment

The preoperative clinical characteristics collected for all enrolled patients included age, sex, body mass index, ethnicity, and history of diabetes, hypertension, and hyperthyroidism.

All preoperative thyroid ultrasounds were performed by experienced sonographers to evaluate nodule characteristics. The ultrasound features assessed included tumor size (≤1.0 cm vs. 1–2 cm vs. 2–4 cm), nodule number (single vs. multiple), tumor location (upper/middle vs. lower), composition (solid vs. non-solid), aspect ratio (≥1 vs. <1), margin (clear vs. unclear), blood flow (poor vs. rich), echogenicity (hypoechoic vs. non-hypoechoic), capsular invasion (no vs. yes), calcification (no calcification vs. microcalcification vs. other types), central lymph node metastasis (unilateral vs. bilateral), and lymph node calcification (no vs. yes).

### Surgical Classification and Management

This study included previously untreated patients with preoperatively low-risk cN1a PTC. Patients were classified into the lobectomy or total thyroidectomy group according to their initial surgical procedure. Patients with intraoperative findings indicative of high-risk features, such as extrathyroidal extension or tumor size >4 cm, were excluded from the cohort to allow us to focus exclusively on low-risk cN1a PTC. The choice between total thyroidectomy and lobectomy solely depends on the number of central lymph node metastases. In cases where pathology revealed hv-CLNM, completion thyroidectomy was not routinely performed. Instead, these patients were managed with regular follow-up. For the recurrence-free survival (RFS) analysis, patients were categorized according to their initial surgical procedure (lobectomy or total thyroidectomy), ensuring clear delineation between groups and minimizing confounding in outcome assessments.

### Histopathological Evaluation

Postoperative histopathological diagnoses were independently evaluated and confirmed by senior pathologists with more than 5 years of experience. All tumor specimens were classified according to the World Health Organization classification of thyroid tumors.

### Postoperative Management and Follow-Up

Follow-up data were obtained via outpatient visits or telephone interviews. Patients underwent regular postoperative follow-up, including Tg and anti-Tg antibody testing, neck ultrasound, or computed tomography. Recurrence was defined as either locoregional (cervical lymph nodes or thyroid bed) or distant (other organs) and confirmed by histological or radiological findings. RFS was defined as the interval between the date of primary thyroid surgery and the date of confirmed recurrence.

### Statistics

Categorical variables were presented as frequencies and percentages (n [%]), and continuous variables were expressed as mean ± standard deviation (x̄ ± s). Group comparisons for categorical variables were performed using Pearson’s chi-squared test or Fisher’s exact test, as appropriate.

Feature selection was initially conducted using the least absolute shrinkage and selection operator (LASSO) regression. The optimal regularization parameter (λ) was determined via 10-fold cross-validation, and the λ value corresponding to the most parsimonious model within one standard error of the minimum cross-validation error (λ_1se) was selected to achieve a balance between model simplicity and explanatory power. In parallel, variables with P < 0.05 in univariate analysis were included in a multivariate logistic regression, with backward stepwise selection based on the Akaike information criterion. As the variables identified by both LASSO and stepwise regression were consistent, a final multivariate logistic regression model was constructed using these variables. A nomogram was then developed based on this model to predict the risk of hv-CLNM in patients with cN1a PTC.

Model performance was assessed by receiver operating characteristic ROC curve analysis, with area under the plasma concentration-time curve (AUC) as the quantitative metric. Model calibration was assessed using the Hosmer–Lemeshow goodness-of-fit test and calibration plots to examine the agreement between predicted and observed outcomes. Internal validation was performed using the bootstrap method with 1000 resampling iterations to evaluate the model’s stability and accuracy. Additionally, within each subgroup, the AUC, sensitivity, and specificity were calculated, and the DeLong test was used to compare AUCs across subgroups to assess potential heterogeneity in model performance.

Additionally, to investigate the prognostic impact of surgical approach, RFS was compared between patients undergoing lobectomy and those undergoing total thyroidectomy. A 1:1 propensity score matching (PSM) approach was applied to adjust for potential confounding factors and balance baseline characteristics between groups. Kaplan–Meier survival curves were constructed to compare RFS between the matched cohorts.

All analyses were performed using R version 4.3.1.

### Results

In this retrospective study, hv-CLNM was histologically confirmed in 13.5% of patients (96/707). The cohort had a mean age of 37.68 ± 10.39 years (range 18–71) and consisted of 569 females (80.5%) and 138 males (19.5%). The average maximum tumor size was 12.38 ± 6.71 mm. Primary tumors were predominantly located in the middle and upper regions of the thyroid gland (465/707 [65.8%]). Concurrent Hashimoto’s thyroiditis (HT), a notable autoimmune comorbidity, was observed in 15.6% of patients (110/707). Ultimately, 332 patients underwent lobectomy and 375 underwent total thyroidectomy (Table 1).

**Table 1:** Clinical and US features

Characteristic	hv-CLNM(-) (N=611)	hv-CLNM(+) (N=96)	*P-value*
*Age, years*			0.004
<45	452 (74.0)	84 (87.5)	
≥45	159 (26.0)	12 (12.5)	
*Sex*			<0.001
Male	100 (16.4)	38 (39.6)	
Female	511 (83.6)	58 (60.4)	
*Ethnicity*			0.651
Han	602 (98.5)	94 (97.9)	
Non-Han	9 (1.5)	2 (2.1)	
*BMI*			0.432
<18.5	59 (9.7)	11 (11.5)	
≥18.5 and ≤24.9	446 (73.0)	64 (66.7)	
>24.9	106 (17.3)	21 (21.9)	
*Diabetes*			1
No	599 (98.0)	95 (99.0)	
Yes	12 (2.0)	1 (1.0)	
*Hypertension*			0.417
No	582 (95.3)	94 (97.9)	
Yes	29 (4.7)	2 (2.1)	
*Hyperthyroidism*			0.618
No	602 (98.5)	96 (100)	
Yes	9 (1.5)	0 (0)	
*Hashimoto’s thyroiditis*			0.135
No	511 (83.6)	86 (89.6)	
Yes	100 (16.4)	10 (10.4)	
*Nodule number*			0.76
Single	308 (50.4)	50 (52.1)	
Multiple	303 (49.6)	46 (47.9)	
*Tumor size, cm*			<0.001
≤1	305 (49.9)	29 (30.2)	
1–2	240 (39.3)	42 (43.8)	
2–4	66 (10.8)	25 (26.0)	
*Tumor location*			<0.001
Middle and upper	417 (68.2)	48 (50.0)	
Lower	194 (31.8)	48 (50.0)	
*Echogenicity*			0.277
Hypoechoic	545 (89.2)	82 (85.4)	
Non-hypoechoic	66 (10.8)	14 (14.6)	
*Aspect ratio*			0.167
<1	291 (47.6)	53 (55.2)	
≥1	320 (52.4)	43 (44.8)	
*Composition*			0.22
Solid	574 (93.9)	87 (90.6)	
No-solid	37 (6.1)	9 (9.4)	
*Calcification*			0.661
No calcification	167 (27.3)	22 (22.9)	
Microcalcification	349 (57.1)	58 (60.4)	
Other types of calcification	95 (15.5)	16 (16.7)	
*Margin*			0.163
Clear	222 (36.3)	42 (43.8)	
Unclear	389 (63.7)	54 (56.3)	
*Blood flow*			0.023
Poor	398 (65.1)	51 (53.1)	
Rich	213 (34.9)	45 (46.9)	
*Capsular invasion*			0.935
No	549 (89.9)	86 (89.6)	
Yes	62 (10.1)	10 (10.4)	
*Central lymph node metastasis*			0.539
Unilateral	493 (80.7)	80 (83.3)	
Bilateral	118 (19.3)	16 (16.7)	
*Lymph node calcification*			<0.001
No	550 (90.0)	73 (76.0)	
Yes	61 (10.0)	23 (24.0)	
*Surgical extent*			0.101
Lobectomy	332 (54.3)	43 (44.8)	
Total thyroidectomy	279 (45.7)	53 (55.2)	
*Lymph node dissection*			0.007
Unilateral	534 (87.4)	74 (77.1)	
Bilateral	77 (12.6)	22 (22.9)	

Data are presented as n (%) unless otherwise indicated

BMI, body mass index; hv-CLNM, high-volume central lymph node metastasis

**Fig. 2:**
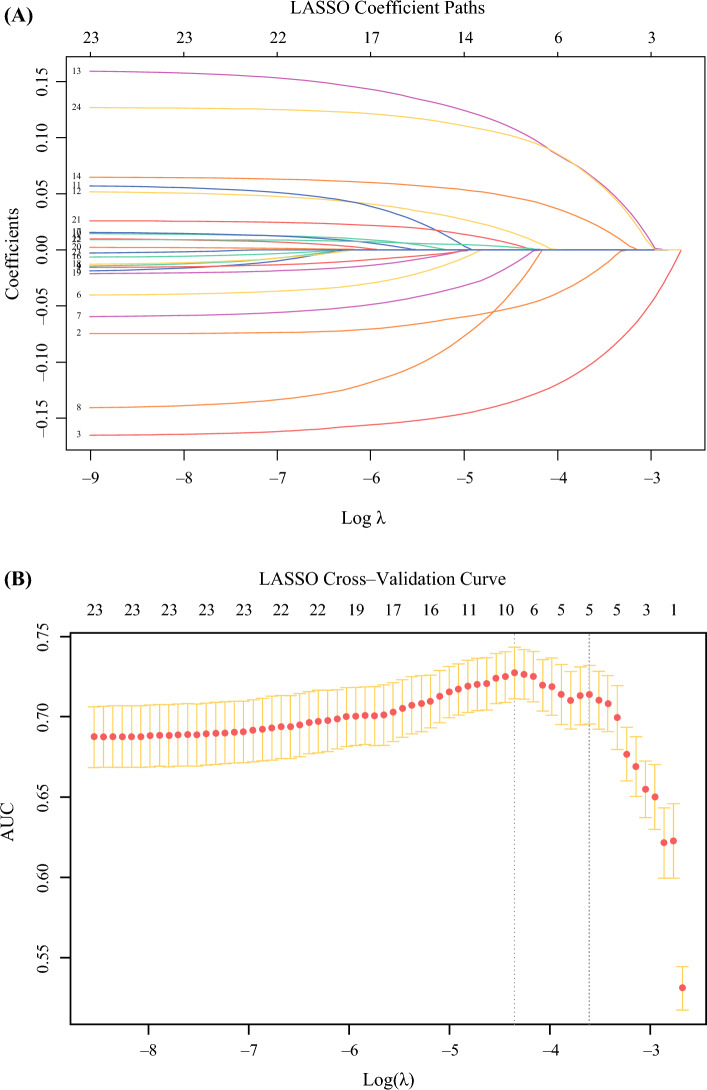
Identification of the influencing factors by least absolute shrinkage and selection operator (LASSO) regression. (a) LASSO coefficient profiles of the selected predictors; (b) ten-fold cross-validation curve for LASSO model using area under the plasma concentration-time curve (AUC) as the performance metric

**Fig. 3:**
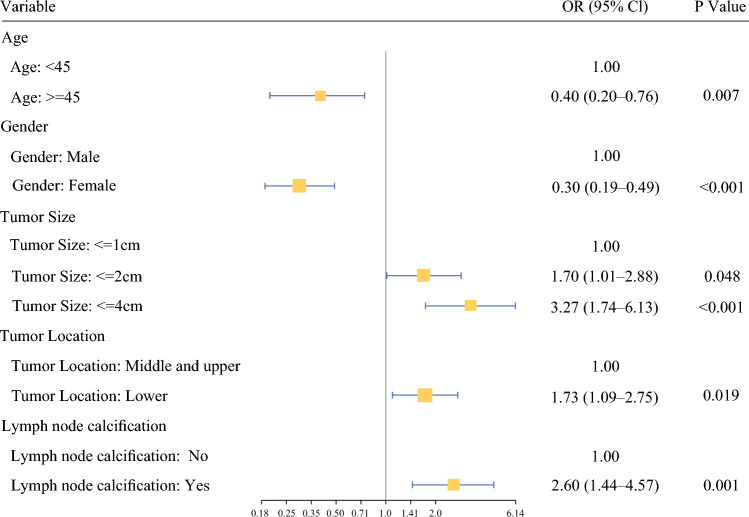
Forest plot of multivariate logistic regression analysis. CI, confidence interval; OR, odds ratio

LASSO regression with 10-fold cross-validation was used for feature selection, with λ₁se chosen for parsimony (Figure 2). Significant variables (*P* < 0.05) from univariate analysis were entered into a multivariate logistic regression using backward stepwise AIC selection (Supplementary Tables S1–S2). Both approaches consistently identified the same set of significant predictors for hv-CLNM: age ≥45 years (odds ratio [OR] 0.40,* P* = 0.007), female (OR 0.30, *P* < 0.001), tumor size 1–2 cm (OR 1.70, *P* = 0.048), tumor size 2–4 cm (OR 3.27, *P* < 0.001), lower pole tumor location (OR 1.73,* P* = 0.019), and the presence of lymph node calcification (OR 2.60, *P* = 0.001) (Figure 3). Given that the enrolled cohort was relatively young, we reconstructed prediction models separately for patients aged ≥45 and <45 years and compared the effect sizes (ORs) of each variable using a forest plot (Supplementary Figure S1).

**Fig. 4:**
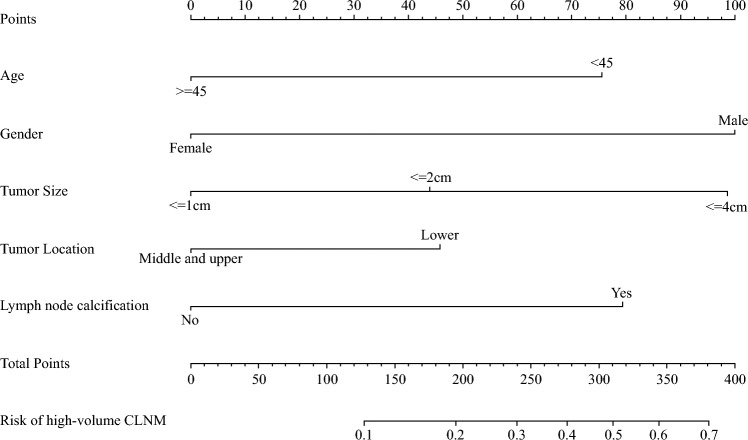
Nomogram to predict high-volume central lymph node metastasis (CLNM)

A nomogram was subsequently constructed incorporating these 5 variables (Fig. 4). For each patient, the corresponding value on the nomogram was scored, and the total score was mapped to the predicted risk of hv-CLNM.

A nomogram was subsequently constructed incorporating these five variables (Fig. 4). For each patient, the corresponding value on the nomogram was scored, and the total score was mapped to the predicted risk of hv-CLNM.

**Fig. 5:**
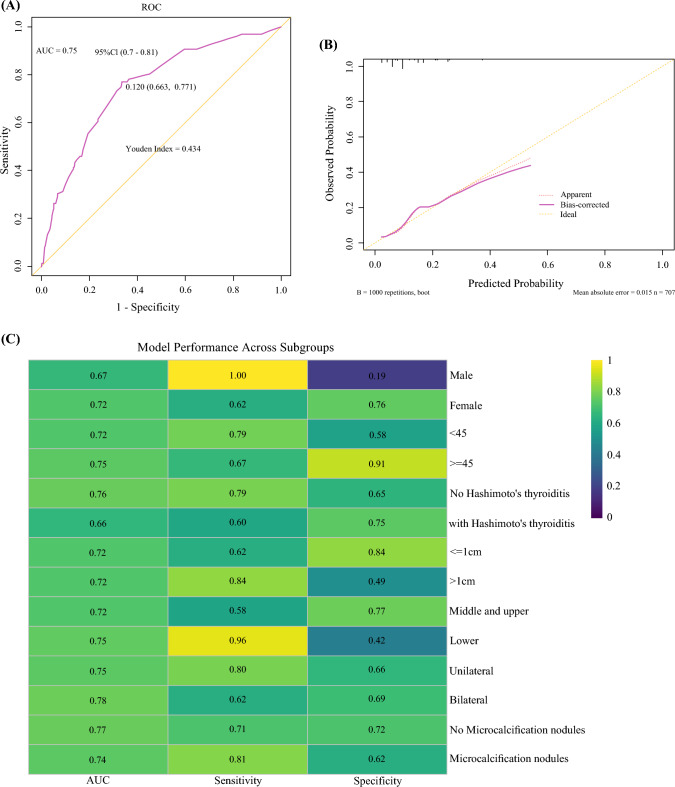
Evaluation of the performance of the prediction model. (a) The receiver operating characteristic (ROC) curve and area under the plasma concentration-time curve (AUC) of the nomogram. (b) Calibration plots of the nomogram for predicting high-volume central lymph node metastasis (hv-CLNM). (c) Model performance across subgroups

The nomogram demonstrated favorable discrimination, with an AUC of 0.75 (95% CI 0.70–0.81) (Fig. 5). The Brier score was 0.107, suggesting low overall prediction error and good model calibration. The calibration slope was 0.918 (95% CI 0.719–1.170), and the Hosmer–Lemeshow test yielded a *P*-value > 0.05, indicating no significant discrepancy between predicted and observed probabilities (Supplementary Table S3).

To evaluate the consistency of model performance across clinically relevant subgroups, we conducted stratified analysis based on the following variables: sex, age (<45 vs. ≥45 years), presence of HT, tumor size (≤1 vs. >1 cm), tumor location (middle/upper vs. lower), laterality of CLNM (unilateral vs. bilateral), and presence of microcalcifications. The model demonstrated stable discriminatory capacity, with AUCs consistently above 0.6 (range 0.66–0.78), and 78.6% (11/14) of subgroups achieving AUCs >0.7 (Figure 5). DeLong’s test for pairwise comparison revealed no statistically significant differences between subgroup AUCs (all *P* > 0.05). These findings indicate good consistency of model performance across subpopulations, suggesting strong generalizability and a low risk of subgroup-specific bias.

To further explore the potential association with HT, we constructed a model in which HT was forcibly included along with the variables selected by LASSO regression. However, the inclusion of HT did not improve predictive performance; key evaluation metrics showed negligible improvement or a slight decline (Supplementary Figures S2–S5).

**Fig. 6:**
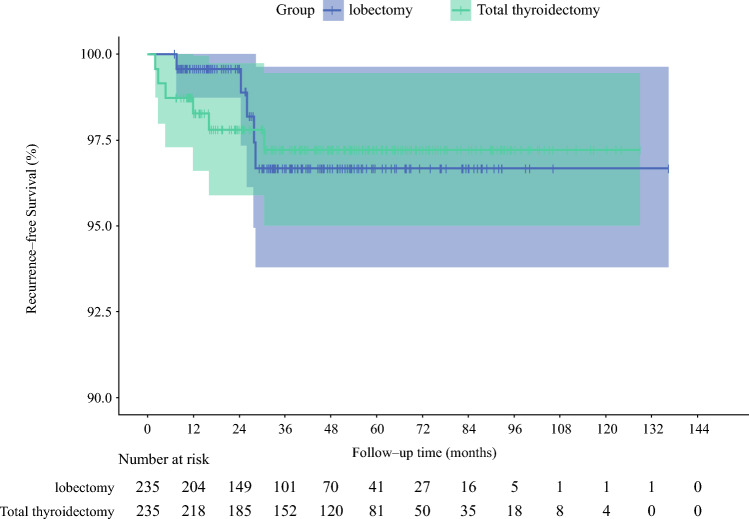
Comparison of recurrence-free survival after propensity score matching

To compare oncologic outcomes, 1:1 PSM was conducted based on age, sex, tumor size, number of metastatic lymph nodes, multifocality, and lymph node ratio. Kaplan–Meier analysis revealed no statistically significant difference in RFS between the lobectomy and total thyroidectomy groups (Fig. 6), indicating that a more conservative surgical approach may be sufficient.

## Discussion

With the growing public awareness of routine health screenings, advancements in imaging modalities such as ultrasonography, and the increased utilization of fine-needle aspiration biopsy, the incidence of PTC has markedly risen over the past 2 decades.^12^ Although the overall prognosis for most patients with PTC is favorable, a subset of individuals remains at higher risk for recurrence and distant metastasis.^13^ Notably, patients with hv-CLNM exhibit a 5-fold increased risk of recurrence compared with those with low-volume metastasis and show a significant association with distant metastases,^14,15^ underscoring the importance of early identification and appropriate aggressive treatment of hv-CLNM.

Current clinical guidelines from the 2015 American Thyroid Association Management Guidelines for Adult Patients with Thyroid Nodules and Differentiated Thyroid Cancer and National Comprehensive Cancer Network Clinical Practice Guidelines in Oncologyrecommend thyroid lobectomy as an initial surgical approach for low-risk patients without aggressive disease features.^10^ Although imaging and clinical history can often exclude overt risk factors such as distant metastases or familial predisposition, assessing central nodal burden preoperatively remains challenging because of the limited sensitivity of ultrasonography.

To address this clinical gap, we enrolled patients with preoperatively low-risk cN1a PTC. Based on preoperative clinical and ultrasonographic data, we developed a predictive model for hv-CLNM. This study identified age, sex, tumor size, tumor location, and lymph node calcification as independent predictors of hv-CLNM. The resulting model demonstrated robust predictive performance. The AUC was 0.75, indicating a moderately high discriminative ability. The calibration slope was 0.87, the Hosmer–Lemeshow goodness-of-fit test yielded a p-value of 0.337, and the Brier score was 0.112, all suggesting good agreement between predicted probabilities and actual outcomes. The model slightly underestimated predicted probabilities, likely because of the exclusion of high-risk patients during its development. The model still demonstrated significant clinical value in predicting hv-CLNM, serving as a useful tool for guiding surgical decision-making and reducing overtreatment in low-risk cN1a cases while ensuring adequate therapy for higher-risk cases.

The overall prevalence of hv-CLNM in this cohort was 13.4%, aligning with previous studies,^16,17^ and remains relatively low. Given the inherent limitations of preoperative ultrasonography in detecting malignant thyroid nodules, it is often challenging to fully assess contralateral occult lesions. Total thyroidectomy, as a radical surgical approach, can potentially eliminate undetected contralateral carcinoma. Among the 332 patients who underwent total thyroidectomy, postoperative pathological analysis revealed 36 cases of contralateral occult carcinoma, accounting for 10.8%. Further pathological examination showed that all these lesions were ≤1 cm in diameter. According to current clinical guidelines,^7,18,19^ such small lesions may be managed with active surveillance rather than immediate surgical intervention. Notably, of these 36 patients, only 5 (13.9%) exhibited hv-CLNM, a proportion similar to that of the overall cohort. Moreover, capsular invasion was identified on postoperative pathology in just 3 of the 36 cases.

The classification of patients based on their initial surgical procedure (lobectomy or total thyroidectomy) and the decision to manage hv-CLNM cases with regular follow-up rather than completion thyroidectomy have important implications for the RFS analysis. By categorizing patients according to their initial surgery, we ensured that the RFS comparison reflected the outcomes of the intended surgical strategy, minimizing potential confounding from subsequent interventions. Our PSM analysis, which included lymph node ratio as a covariate, further mitigated potential biases arising from differences in lymph node dissection extent or metastatic burden between the lobectomy and total thyroidectomy groups. After PSM, no significant difference in RFS was observed between lobectomy and total thyroidectomy groups. This aligns with studies in cN1b patients by Xu et al.^20^ and Saito et al.^21^. These results suggest that lobectomy may be a safe and sufficient option for most patients with cN1a PTC without other risk features. However, the relatively small number of hv-CLNM cases in the lobectomy group (43/375 [11.5%]) warrants further investigation in larger cohorts to confirm the generalizability of these results.

Age has long been recognized as a critical prognostic factor in patients with PTC.^22,23^ In our study, patients aged >45 years were less likely to have hv-CLNM, consistent with prior evidence that older age may confer a protective effect.^24^ The mean age of the cohort (37.68 years) is notably younger than that observed in most large-scale studies of thyroid cancer, and the sample size of patients aged ≥45 years (n=171) was relatively small, with only 12 cases of hv-CLNM, which may introduce some bias into the model’s performance for this subgroup. So, we reconstructed the model separately in subgroups of age <45 years and ≥45 years and displayed the OR changes of variables using a forest plot. In the group aged <45 years, female sex was a protective factor (OR 0.28, P < 0.001), whereas larger tumor size (>2 cm), lower pole tumor location, and lymph node calcification were significantly associated with an increased risk of hv-CLNM. In contrast, among patients aged ≥45 years, the predictive effects of sex and tumor size were attenuated and did not reach statistical significance. Notably, lymph node calcification remained a strong predictor across both age groups, with an even more pronounced risk effect in older patients (OR 7.19, P = 0.007). Although the risk factors for hv-CLNM showed some differences between the two age groups, the model consistently exhibited good discriminative ability, with an AUC of 0.72 in patients aged <45 years and 0.75 in those aged ≥45 years. These findings suggest that the predictive model is robust across age strata and may be applicable to a broader patient population. In future studies with larger sample sizes, the risk factors associated with hv-CLNM in younger and older patients can be further explored and distinguished in greater detail.

Additionally, a wealth of evidence indicates that, although PTC is more prevalent in women, they tend to have lower rates of CLNM, distant metastasis, and cancer-specific mortality.^25^ In this cohort, the male-to-female ratio among CLNM patients was approximately 1:5, and further analysis confirmed that female sex was an independent protective factor for CLNM (OR 0.30, *P* < 0.001). Therefore, treatment strategies may need to be individualized according to age and sex: elderly female patients may be more suitable for lobectomy, whereas younger male patients could benefit from more aggressive interventions such as total thyroidectomy.^26^

Tumor location is closely associated with lymph node metastasis in PTC. For lateral neck lymph node metastasis, tumors located in the upper portion of the thyroid lobe have been identified as a significant risk factor.^27,28^ In contrast, for central compartment lymph node metastasis, tumors situated in the lower portion of the thyroid are more likely to be involved.^29^ Our results support this pattern, showing that tumors located in the lower third of the thyroid were more likely to be associated with hv-CLNM.

HT is the most common autoimmune thyroid disorder.^30^ Since Dailey et al.^31^ first described a possible association between HT and PTC in 1955, this relationship has remained controversial. Some studies have suggested that HT may play a protective role, potentially through enhanced immune surveillance and anti-tumor antibody production.^32^ Specifically, several reports have proposed that HT may reduce the risk of hv-CLNM,^12^ whereas others have found no significant correlation.^24^ In our study, univariate analysis revealed no significant association between HT and hv-CLNM (p = 0.135). Furthermore, adding HT to the multivariate model did not improve predictive performance. These results suggest that HT is not an independent predictor of hv-CLNM in our cohort. Further studies with larger cohorts and stratified analyses may help clarify whether HT plays a role in specific subgroups of patients with PTC.

In our predictive model for hv-CLNM in patients with cN1a PTC, lymph node calcification emerged as an independent risk factor. The presence of calcified lymph nodes on ultrasound is often considered one of the malignant indicators suggestive of lymph node metastasis.^33^ Although most studies have focused on the impact of nodule calcification on hv-CLNM,^34^ few have specifically investigated the predictive value of lymph node calcification. Our findings highlight the potential value of lymph node calcification as an ultrasound feature of tumor metastatic burden in patients with cN1a PTC.

## Limitations

This study has several limitations that warrant further investigation in future research. First, this was a single-center retrospective study with a relatively small sample size, and so selection bias is inevitable. Second, external validation is lacking, so prospective, multicenter clinical studies are needed to further assess the robustness and generalizability of the proposed model. Large-scale, multicenter prospective studies are recommended to enhance the reliability of the findings. Third, the evaluation of certain ultrasound features is inherently subjective and may be affected by interobserver variability, potentially compromising the consistency of the results.

## Conclusions

Although most prior research has focused on the presence or absence of CLNM or the risk factors for hv-CLNM in clinically node-negative (cN0) PTC,^35,36^ few have addressed this issue in clinically evident cN1a disease. To our knowledge, this is the first study to explore hv-CLNM in preoperatively low-risk cN1a PTC and build a predictive model based on clinical and ultrasound features. This model we developed provides a practical tool for guiding the extent of initial surgery in patients with cN1a PTC. By identifying those at low risk for hv-CLNM, clinicians may avoid unnecessary total thyroidectomy and its associated risks. Our approach promotes individualized, risk-adapted surgical planning and helps minimize overtreatment in this growing population of low-risk patients.

Age, sex, tumor size, tumor location, and lymph node calcification were identified as significant predictors of hv-CLNM. Based on these variables, we developed a nomogram that integrates clinical and ultrasonographic features to predict the risk of hv-CLNM, thereby assisting clinicians in making individualized treatment decisions. Notably, there was no significant difference in prognosis between lobectomy and total thyroidectomy in these patients. Lobectomy may be a more appropriate surgical approach for the majority of patients within this subgroup.

## Supplementary Information

Below is the link to the electronic supplementary material.

Supplementary file 1 (DOCX 13 KB)

Supplementary file 2 (DOCX 13 KB)

Supplementary file 3 (DOCX 12 KB)

Supplementary file 4 (JPG 406 KB)

Supplementary file 5 (JPG 530 KB)

Supplementary file 6 (JPG 315 KB)

Supplementary file 7 (JPG 326 KB)

Supplementary file 8 (JPG 418 KB)

## Data Availability

The datasets produced in this study are available upon reasonable request by reaching out to the corresponding.

## References

[1] Lim H, et al. Trends in Thyroid Cancer Incidence and Mortality in the United States, 1974–2013. *JAMA*. 2017;317(13):1338–48. 10.1001/jama.2017.2719. 28362912 10.1001/jama.2017.2719 PMC8216772

[2] Londero SC, et al. Papillary thyroid carcinoma in Denmark, 1996–2008: outcome and evaluation of established prognostic scoring systems in a prospective national cohort. *Thyroid*. 2015;25(1):78–84. 10.1089/thy.2014.0294. 25368981 10.1089/thy.2014.0294

[3] Zhao H, Li H. Meta-analysis of ultrasound for cervical lymph nodes in papillary thyroid cancer: Diagnosis of central and lateral compartment nodal metastases. *Eur J Radiol*. 2019;112:14–21. 10.1016/j.ejrad.2019.01.006. 30777203 10.1016/j.ejrad.2019.01.006

[4] Matsuzu K, et al. Thyroid lobectomy for papillary thyroid cancer: long-term follow-up study of 1,088 cases. *World J Surg*. 2014;38(1):68–79. 10.1007/s00268-013-2224-1. 24081532 10.1007/s00268-013-2224-1

[5] Cao M, et al. The preferred surgical choice for intermediate-risk papillary thyroid cancer: total thyroidectomy or lobectomy? A systematic review and meta-analysis. *Int J Surg*. 2024;110(8):5087–100. 10.1097/JS9.0000000000001556. 38967517 10.1097/JS9.0000000000001556 PMC11325972

[6] Kovatch KJ, Hoban CW, Shuman AG. Thyroid cancer surgery guidelines in an era of de-escalation. *Eur J Surg Oncol*. 2018;44(3):297–306. 10.1016/j.ejso.2017.03.005. 28385370 10.1016/j.ejso.2017.03.005 PMC5600641

[7] Haddad RI, Bischoff L, Ball D, Bernet V, Blomain E, Busaidy NL, Campbell M, Dickson P, Duh QY, Ehya H, Goldner WS. Thyroid carcinoma, version 2 2022, NCCN clinical practice guidelines in oncology. *J National Comprehensive Cancer Network*. 2022;20(8):925–51. 10.6004/jnccn.2022.0040 35948029

[8] Pérez-Soto RH, et al. Preoperative and Postoperative Risk Stratification of Thyroid Papillary Microcarcinoma: A Comparative Study Between Kuma Criteria and 2015 American Thyroid Association Guidelines Risk Stratification. *Thyroid*. 2020;30(6):857–62. 10.1089/thy.2019.0698. 32031061 10.1089/thy.2019.0698

[9] Lee J, Song Y, Soh EY. Prognostic significance of the number of metastatic lymph nodes to stratify the risk of recurrence. *World J Surg*. 2014;38(4):858–62. 10.1007/s00268-013-2345-6. 24305921 10.1007/s00268-013-2345-6

[10] Haugen BR, et al. 2015 American Thyroid Association Management Guidelines for Adult Patients with Thyroid Nodules and Differentiated Thyroid Cancer: The American Thyroid Association Guidelines Task Force on Thyroid Nodules and Differentiated Thyroid Cancer. *Thyroid*. 2016;26(1):1–133. 10.1089/thy.2015.0020. 26462967 10.1089/thy.2015.0020 PMC4739132

[11] Xing Z, et al. Thyroid cancer neck lymph nodes metastasis: Meta-analysis of US and CT diagnosis. *Eur J Radiol*. 2020;129:109103. 10.1016/j.ejrad.2020.109103. 32574937 10.1016/j.ejrad.2020.109103

[12] Ugai T, et al. Is early-onset cancer an emerging global epidemic? Current evidence and future implications. *Nat Rev Clin Oncol*. 2022;19(10):656–73. 10.1038/s41571-022-00672-8. 36068272 10.1038/s41571-022-00672-8 PMC9509459

[13] Ito Y, et al. Patient age is significantly related to the progression of papillary microcarcinoma of the thyroid under observation. *Thyroid*. 2014;24(1):27–34. 10.1089/thy.2013.0367. 24001104 10.1089/thy.2013.0367 PMC3887422

[14] Randolph GW, et al. The prognostic significance of nodal metastases from papillary thyroid carcinoma can be stratified based on the size and number of metastatic lymph nodes, as well as the presence of extranodal extension. *Thyroid*. 2012;22(11):1144–52. 10.1089/thy.2012.0043. 23083442 10.1089/thy.2012.0043

[15] Machens A, Dralle H. Correlation between the number of lymph node metastases and lung metastasis in papillary thyroid cancer. *J Clin Endocrinol Metab*. 2012;97(12):4375–82. 10.1210/jc.2012-1257. 23019347 10.1210/jc.2012-1257

[16] Huang X, Gan X, Feng J, Cai W, Xu B. Nomograms for predicting cervical central lymph node metastases and high-volume cervical central lymph node metastases in papillary thyroid carcinoma. *Gland Surg*. 2025;14(3):421. 40256478 10.21037/gs-24-237 PMC12004298

[17] Wei X, et al. Development and validation of an individualized nomogram for predicting the high-volume (> 5) central lymph node metastasis in papillary thyroid microcarcinoma. *J Endocrinol Invest*. 2022;45(3):507–15. 10.1007/s40618-021-01675-5. 34491546 10.1007/s40618-021-01675-5

[18] Sugitani I, et al. The 2024 revised clinical guidelines on the management of thyroid tumors by the Japan Association of Endocrine Surgery. *Endocr J*. 2025;72(5):545–635. 10.1507/endocrj.EJ24-0644. 40058844 10.1507/endocrj.EJ24-0644 PMC12086281

[19] Liu S, Xu Z, Wang P, Wang Y, Zhu Y, Sun H, Yang A, He X, Lin Y, Yi J, Luo D. National guidelines for diagnosis and treatment of thyroid cancer 2022 in China (English version) National Health Commission of the People’s Republic of China. *Chinese J Cancer Res*. 2022;34(3):131–50. 10.21147/j.issn.1000-9604.2022.03.01 PMC9273579 35873884

[20] Xu S, et al. Comparison of lobectomy vs total thyroidectomy for intermediate-risk papillary thyroid carcinoma with lymph node metastasis. *JAMA Surg*. 2023;158(1):73–9. 10.1001/jamasurg.2022.5781. 36449303 10.1001/jamasurg.2022.5781 PMC9713681

[21] Saito Y, et al. Lobectomy vs Total Thyroidectomy With Ipsilateral Lateral Neck Dissection for N1b Intermediate-Risk Papillary Thyroid Carcinoma. *JAMA Otolaryngol Head Neck Surg*. 2025;151(2):105–12. 10.1001/jamaoto.2024.3860. 39602155 10.1001/jamaoto.2024.3860 PMC11826362

[22] Siddiqui S, et al. Clinical and Pathologic Predictors of Lymph Node Metastasis and Recurrence in Papillary Thyroid Microcarcinoma. *Thyroid*. 2016;26(6):807–15. 10.1089/thy.2015.0429. 27117842 10.1089/thy.2015.0429

[23] Cho J, et al. Clinical features and prognostic factors in papillary thyroid microcarcinoma depends on age. *J Korean Surg Soc*. 2012;82(5):281–7. 10.4174/jkss.2012.82.5.281. 22563534 10.4174/jkss.2012.82.5.281 PMC3341476

[24] Wang Z, et al. Clinical and ultrasonic risk factors for high-volume central lymph node metastasis in cN0 papillary thyroid microcarcinoma: A retrospective study and meta-analysis. *Clin Endocrinol (Oxf)*. 2023;98(4):609–21. 10.1111/cen.14834. 36263602 10.1111/cen.14834

[25] Shobab L, Burman KD, Wartofsky L. Sex Differences in Differentiated Thyroid Cancer. *Thyroid*. 2022;32(3):224–35. 10.1089/thy.2021.0361. 34969307 10.1089/thy.2021.0361

[26] Wang D, et al. Predictive nomogram for central lymph node metastasis in papillary thyroid microcarcinoma based on pathological and ultrasound features. *Front Endocrinol (Lausanne)*. 2023;14:1108125. 10.3389/fendo.2023.1108125. 37484943 10.3389/fendo.2023.1108125 PMC10358981

[27] Zhao L, et al. Risk factors of skip lateral cervical lymph node metastasis in papillary thyroid carcinoma: a systematic review and meta-analysis. *Endocrine*. 2022;75(2):351–9. 10.1007/s12020-021-02967-9. 35067901 10.1007/s12020-021-02967-9

[28] Feng J, et al. Nomograms for Prediction of High-Volume Lymph Node Metastasis in Papillary Thyroid Carcinoma Patients. *Otolaryngol Head Neck Surg*. 2023;168(5):1054–66. 10.1002/ohn.161. 36856043 10.1002/ohn.161

[29] Zhang TT, Qi XZ, Chen JP, Shi RL, Wen SS, Wang YL, Ji QH, Shen Q, Zhu YX, Qu N. The association between tumor’s location and cervical lymph nodes metastasis in papillary thyroid cancer. *Gland Surg*. 2019;8(5):557. 31741887 10.21037/gs.2019.10.02 PMC6842756

[30] McLeod DSA, Cooper DS. The incidence and prevalence of thyroid autoimmunity. *Endocrine*. 2012;42(2):252–65. 10.1007/s12020-012-9703-2. 22644837 10.1007/s12020-012-9703-2

[31] Dailey ME, Lindsay S, Skahen R. Relation of thyroid neoplasms to Hashimoto disease of the thyroid gland. *AMA Arch Surg*. 1955;70(2):291–7. 10.1001/archsurg.1955.01270080137023. 13227748 10.1001/archsurg.1955.01270080137023

[32] Kim SS, et al. Coexistence of Hashimoto’s thyroiditis with papillary thyroid carcinoma: the influence of lymph node metastasis. *Head Neck*. 2011;33(9):1272–7. 10.1002/hed.21594. 21837696 10.1002/hed.21594

[33] **中国医**师协会外科医师分会甲状腺外科专家工作组与中国研究型医院学会甲状腺疾病专业委员会,**超声引**导下甲状腺结节和颈部淋巴结细针穿刺活检中国专家共识及操作指南（2025**版**）.**中国**实用外科杂志, 2025. 45(01):**第**34-41页.

[34] Liu C, et al. Risk factor analysis for predicting cervical lymph node metastasis in papillary thyroid carcinoma: a study of 966 patients. *BMC Cancer*. 2019;19(1):622. 10.1186/s12885-019-5835-6. 31238891 10.1186/s12885-019-5835-6 PMC6593593

[35] Hu L, et al. Construction and validation of nomograms to predict central lymph node metastasis in clinical node-negative unilateral papillary thyroid carcinoma. *Sci Rep*. 2025;15(1):2662. 10.1038/s41598-025-86201-w. 39837926 10.1038/s41598-025-86201-w PMC11751388

[36] Zhu H, et al. Development and validation of a clinical predictive model for high-volume lymph node metastasis of papillary thyroid carcinoma. *Sci Rep*. 2024;14(1):15828. 10.1038/s41598-024-66304-6. 38982104 10.1038/s41598-024-66304-6 PMC11233634

