# Supplementary material for: Surgical Optimization in Preoperatively Low-risk cN1a PTC: A Predictive Model for High-Volume Central Lymph Node Metastasis
Source: Ann Surg Oncol. 2025 Oct 22;33(2):1307–18. doi: 10.1245/s10434-025-18569-y (PMC12765736; doi:10.1245/s10434-025-18569-y)
Supplement: Supplementary file 2 — (DOCX 13 KB) [file 10434_2025_18569_MOESM2_ESM.docx]

Table S2：Multivariate Logistic Regression with Backward Stepwise Selection Based on AIC

| **Characteristic** | **log(OR)** | **95% CI** | ***p*-value** |
| --- | --- | --- | --- |
| Age |  |  |  |
| <45 | Ref |  |  |
| >=45 | -0.91 | -1.6, -0.28 | 0.007 |
| Gender |  |  |  |
| Male | Ref |  |  |
| Female | -1.2 | -1.7, -0.71 | <0.001 |
| Tumor Size |  |  |  |
| <=1cm | Ref |  |  |
| 1-2cm | 0.53 | 0.01, 1.1 | 0.048 |
| 2-4cm | 1.2 | 0.55, 1.8 | <0.001 |
| Tumor Location |  |  |  |
| Middle and upper | Ref |  |  |
| Lower | 0.55 | 0.09, 1.0 | 0.019 |
| Lymph node calcification |  |  |  |
| No | Ref |  |  |
| Yes | 0.95 | 0.37, 1.5 | 0.001 |
