# Supplementary material for: Surgical Optimization in Preoperatively Low-risk cN1a PTC: A Predictive Model for High-Volume Central Lymph Node Metastasis
Source: Ann Surg Oncol. 2025 Oct 22;33(2):1307–18. doi: 10.1245/s10434-025-18569-y (PMC12765736; doi:10.1245/s10434-025-18569-y)
Supplement: Supplementary file 3 — (DOCX 12 KB) [file 10434_2025_18569_MOESM3_ESM.docx]

Table S3：Model Performance Metrics Estimated by Bootstrap Cross-Validation (1,000 Iterations)

| VAR | Metric | Mean | CI_Lower | CI_Upper |
| --- | --- | --- | --- | --- |
| 1 | AUC | 0.7453240 | 0.7244148843 | 0.7570572 |
| 2 | Sensitivity | 0.7107292 | 0.5208333333 | 0.8750000 |
| 3 | Specificity | 0.6816907 | 0.4991816694 | 0.8166524 |
| 4 | brier score | 0.1065686 | 0.1052964828 | 0.1087860 |
| 5 | Calibrate slope | 0.9181636 | 0.7193824282 | 1.1699968 |
