# Supplementary figures and images for: Surgical Optimization in Preoperatively Low-risk cN1a PTC: A Predictive Model for High-Volume Central Lymph Node Metastasis
Source: Ann Surg Oncol. 2025 Oct 22;33(2):1307–18. doi: 10.1245/s10434-025-18569-y (PMC12765736; doi:10.1245/s10434-025-18569-y)

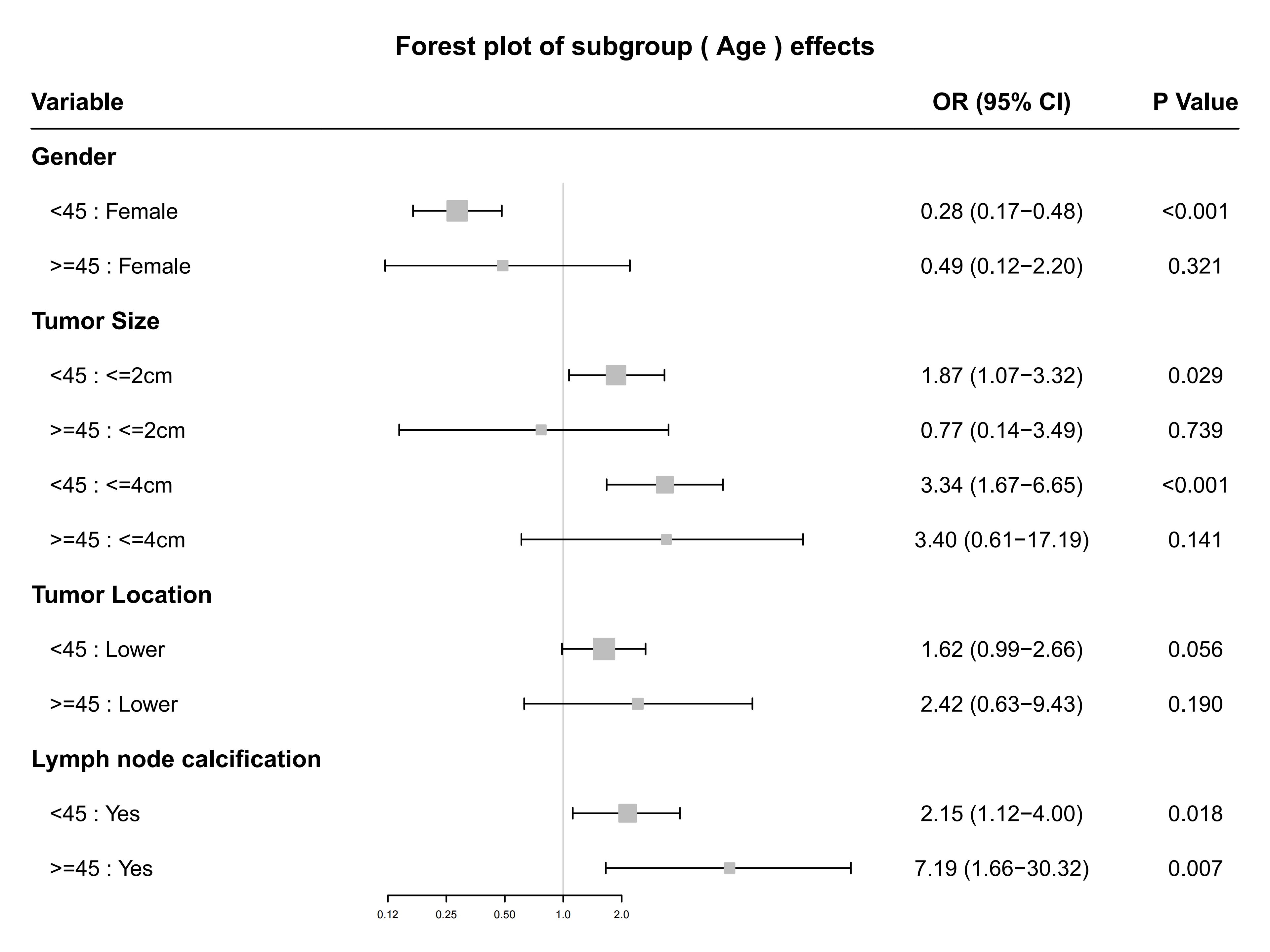

Supplement: Supplementary file 4 — (JPG 406 KB) [file 10434_2025_18569_MOESM4_ESM.jpg]

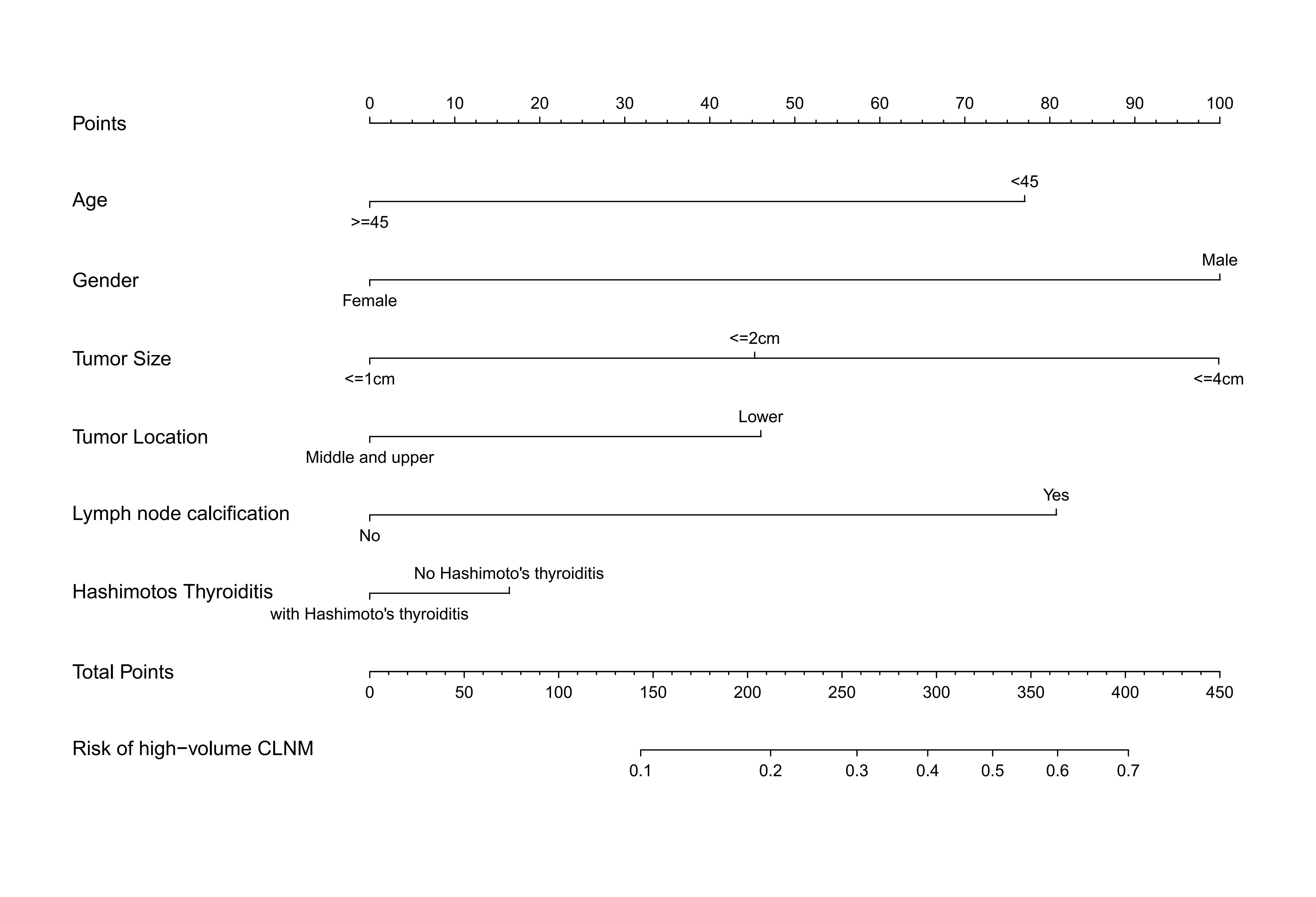

Supplement: Supplementary file 5 — (JPG 530 KB) [file 10434_2025_18569_MOESM5_ESM.jpg]

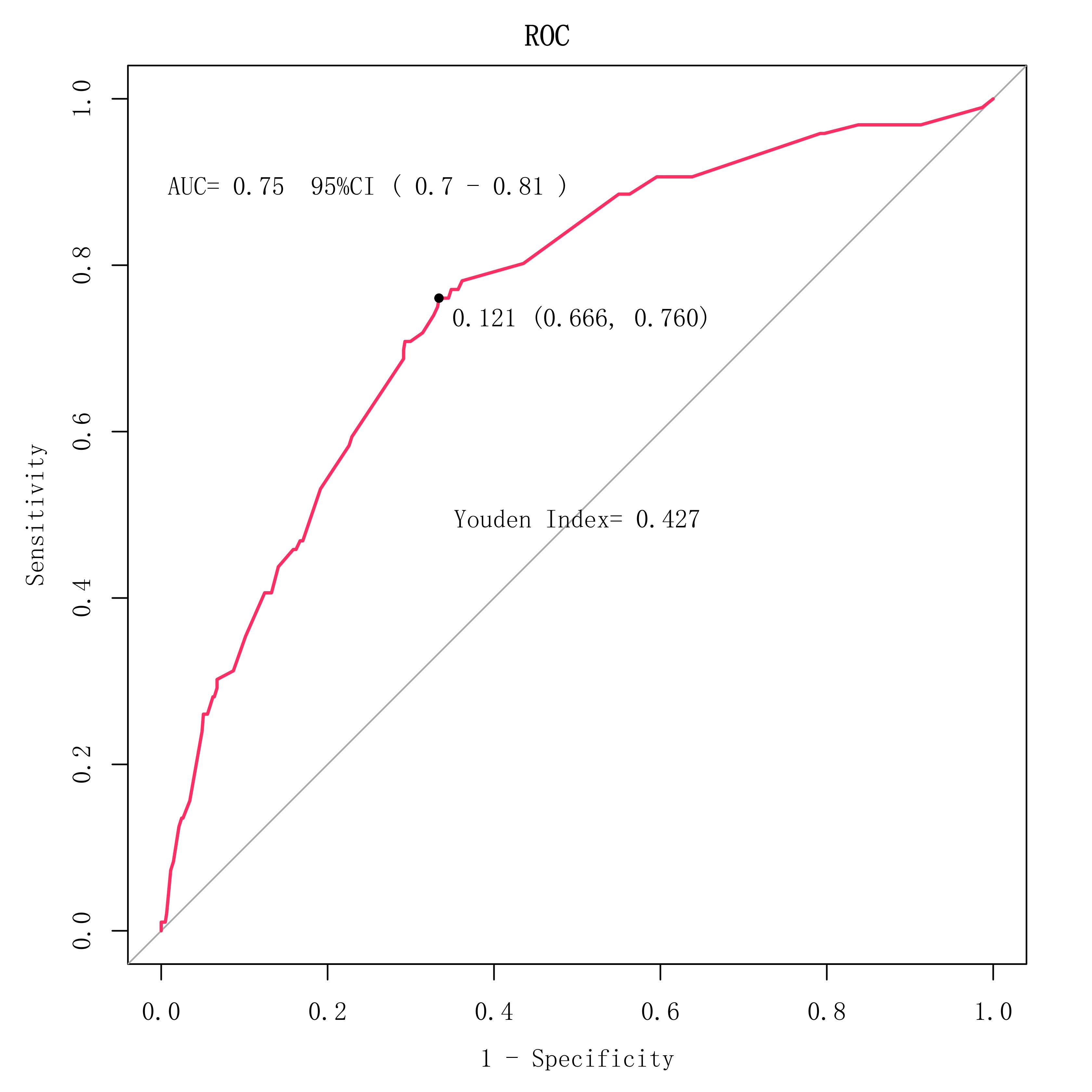

Supplement: Supplementary file 6 — (JPG 315 KB) [file 10434_2025_18569_MOESM6_ESM.jpg]

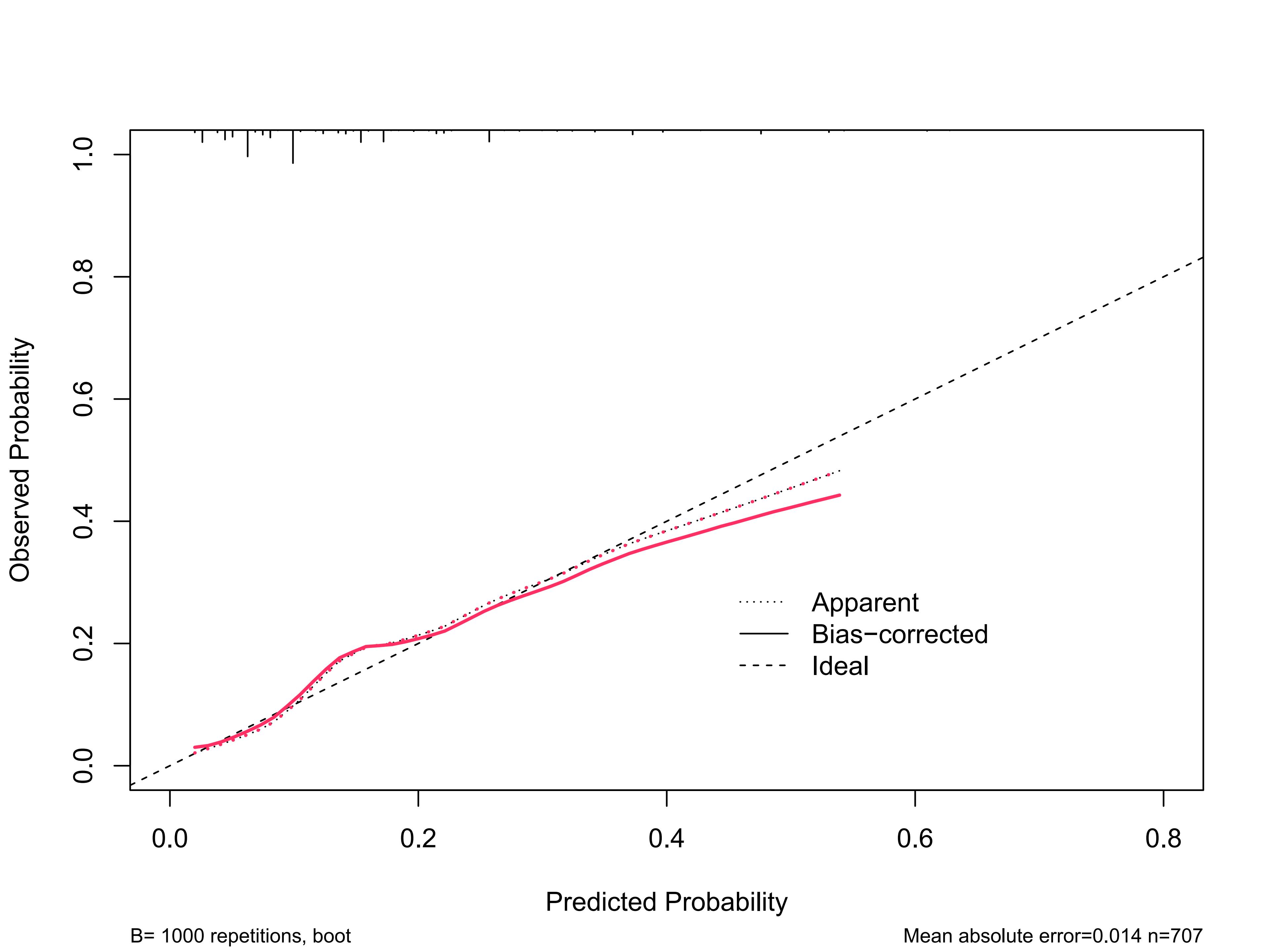

Supplement: Supplementary file 7 — (JPG 326 KB) [file 10434_2025_18569_MOESM7_ESM.jpg]

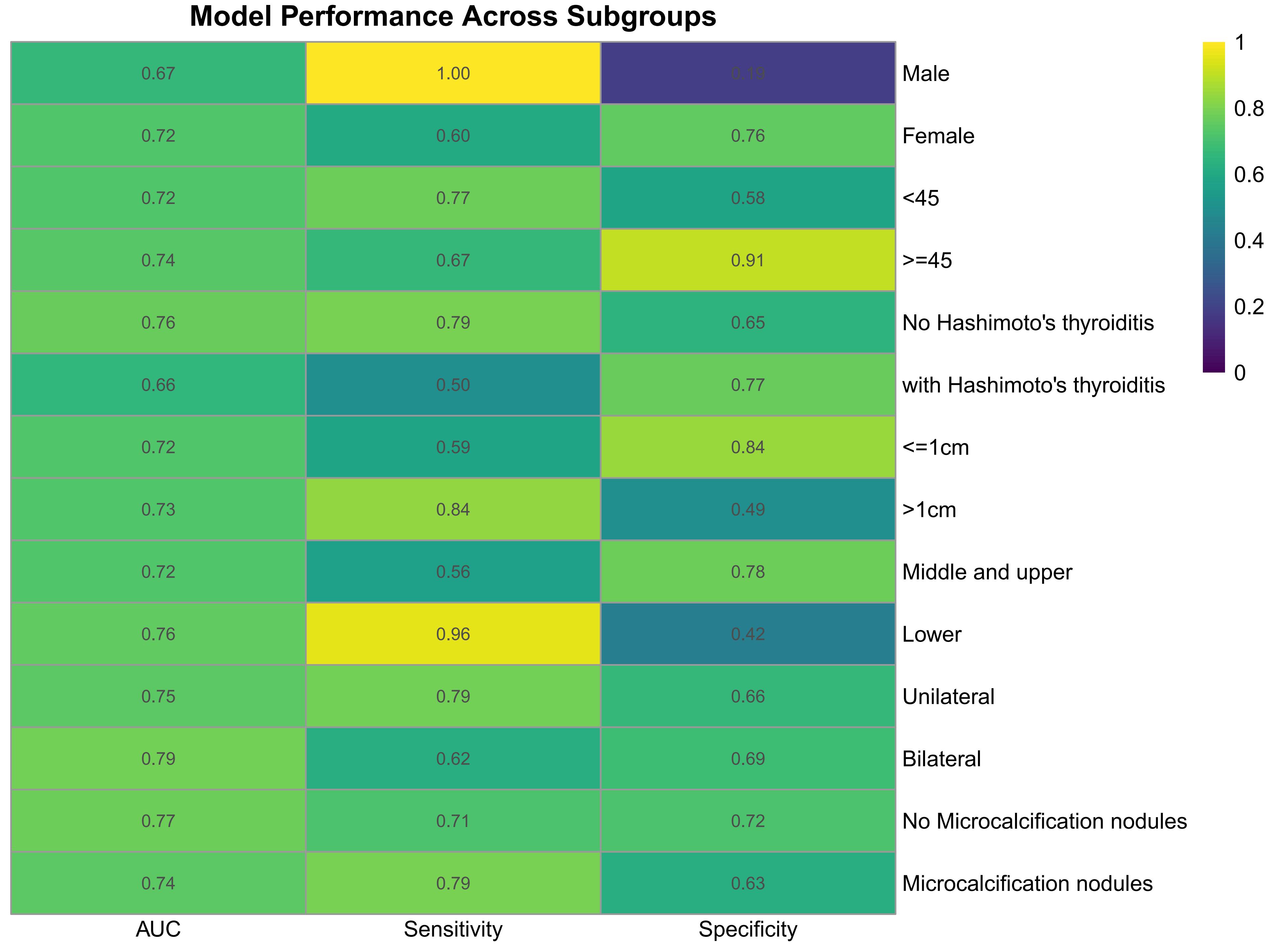

Supplement: Supplementary file 8 — (JPG 418 KB) [file 10434_2025_18569_MOESM8_ESM.jpg]
